# The influence of extreme rainfall on crime: Spatiotemporal dynamics in Hunan Province, China

**DOI:** 10.1371/journal.pone.0345811

**Published:** 2026-04-22

**Authors:** Dongshui Zhang, Yanlu Xiao, Yongshun Han, Tongsheng Li, Xiuquan Li, Xinbao Chen, Jun Fang, Pin Zhong, Zhe Ning

**Affiliations:** 1 School of Earth Sciences and Spatial Information Engineering, Hunan University of Science and Technology, Xiangtan, China; 2 Hunan Institute of Geological Disaster Investigation and Monitoring, Changsha, China; 3 Hunan Geological Disaster Monitoring Early Warning and Emergency Rescue Engineering Technology Research Center, Changsha, China; Universiti Teknologi Malaysia - Main Campus Skudai: Universiti Teknologi Malaysia, MALAYSIA

## Abstract

The influence of extreme weather events on social stability is increasingly acknowledged as a critical and pressing concern. Utilizing data on the criminal offenses, extreme climate conditions, and socio-economic indicators from counties and cities in Hunan Province for the period 2014–2018, this study systematically examines the impact of extreme heavy rainfall events on crime. By integrating a standardized crime intensity index, spatial autocorrelation analysis, grey correlation analysis, and a panel data model, the research comprehensively analyzes the underlying mechanisms and spatiotemporal dynamics of this relationship. The main conclusions are as follows: (1) Heavy rainfall exerts a discernible influence on the spatial distribution of crime, especially in agricultural regions such as Ningxiang and Dongkou. In these areas, the interruption of farming activities caused by intense precipitation appears to increase susceptibility to criminal incidents. (2) Extended rainfall events are associated with elevated crime rates, increasing by approximately 0.374 percent for each 1 percent increase in storm duration. In contrast, variations in rainfall intensity show no substantial influence on crime incidence. (3) Socio-economic conditions, including population mobility, GDP per capita, and the proportion of tertiary industry, further complicate this relationship by amplifying the influence of rainfall under specific contextual settings. The findings provide tailored strategies for preventing crime in regions that are particularly vulnerable to disruptions in agricultural production.

## 1. Introduction

Understanding the influence of extreme weather events, such as heavy rainfall, on social stability is of great significance, particularly in rapidly developing regions [1,2]. The analysis of criminal activity provides valuable insights into overall social stability, government performance, and regional security [2,3]. In recent decades, the increasing frequency of extreme weather events due to global climate change has significantly influenced crime rates, as well as, the surrounding social and environmental conditions [3,4]. Regional variations in socio-economic structures and population mobility further complicate the effects of such events on crime patterns, creating heterogeneous impacts across different areas [5–7]. Therefore, a thorough understanding of how natural environmental changes interact with social factors to shape criminal behavior and its spatial distribution is of considerable practical importance for formulating effective social security strategies.

Against this backdrop, researchers have increasingly endeavored to elucidate the impact of extreme weather on criminal behavior and the broader social environment. While traditional criminological theories emphasize socio-economic determinants, the intensifying impacts of climate change underscore the role of environmental factors in shaping crime patterns. Routine Activity Theory suggests that environmental shocks can increase crime opportunities by disrupting routines [8], whereas General Strain Theory argues that stressors such as economic hardship may elevate criminal behavior [9]. Although recent studies have verified that extreme weather can disrupt social order and affect crime rates [10,11], the underlying mechanisms and the overall nature of this relationship remain incompletely understood. This serves as an impetus for more context-specific, fine-scale studies that explicitly take into account spatial and temporal heterogeneity and determine which dimensions of extreme rainfall (e.g., duration versus intensity) are of the greatest significance for crime outcomes [12,13].

Importantly, empirical evidence indicates that the effects of extreme weather conditions on crime are not consistent; they differ according to the type of crime and are influenced by regional socio-economic structures. For example, research conducted in Taiwan has shown that moderate-intensity typhoons may lead to an increase in motor vehicle theft but a decrease in drug-related crimes [14], while studies in Colombia indicate that floods can result in a decrease in personal theft while potentially causing an increase in burglaries [15]. Moreover, analyses across European regions demonstrate that regional socio-economic structures play a significant moderating role [16,17]. Furthermore, findings from urban climate adaptation research and global scoping reviews suggest that factors such as resource allocation and adaptive capacity can mitigate adverse impacts, while economic instability may exacerbate them [18,19]. Collectively, these findings imply that the relationships between weather and crime are context-dependent. Nevertheless, the majority of existing studies have been carried out at the national aggregate level or in major cities, providing limited evidence regarding fine-scale spatiotemporal patterns in rapidly developing, agriculture-dependent counties and how local socio-economic conditions influence these effects.

Hunan Province provides a suitable case for addressing this gap. Situated in a subtropical monsoon region characterized by complex terrain, the area experiences frequent heavy rainfall [20]. The province also demonstrates significant urban-rural disparities, as numerous counties remain reliant on agriculture while undergoing socio-economic transformation. These characteristics may influence how rainfall-related disruptions manifest as changes in crime opportunities and local vulnerability. However, county-level empirical evidence that specifically quantifies the relationship between extreme rainfall and crime in Hunan is still scarce.

To address these gaps, this study undertakes a systematic investigation of the impact of extreme rainfall on crime at the county level in Hunan Province. Through the utilization of a standardized crime intensity index, spatial autocorrelation, grey correlation analysis, and panel data modeling, it quantifies the association between rainfall and crime, uncovers its spatial heterogeneity, and examines whether local socio-economic conditions moderate this relationship. This research provides novel county-level empirical evidence from an under-studied, agriculture-dependent region, presents a transferable multi-method analytical approach, and offers policy-relevant insights to support climate adaptation and targeted crime-prevention strategies in Hunan and similar transitioning areas.

## 2. Study area and data sources

### 2.1 Study area

Hunan Province is situated in the central region of China, in the middle and lower reaches of the Yangtze River, boasting a total area of approximately 211,800 square kilometers [20–22]. Characterized by a subtropical monsoon climate, Hunan Province features concurrent precipitation and high temperatures, with hot and rainy summers and dry winters. In recent years, Hunan Province has encountered more frequent extreme weather events, including heavy rainfall, intense heat, and floods, which have exerted a significant influence on the local economy and daily life [20–22]. As a key participant in China’s central region’s development strategy, Hunan, especially in the vicinity of the Chang-Zhu-Tan urban cluster, is undergoing rapid urban development. This has led to significant population shifts and a more intricate social environment [20–22]. These features make Hunan a representative region for researching the relationship between extreme climate events and social impacts. Moreover they offer crucial reference significance for comprehending the impacts of global climate change on social stability.

### 2.2 Data sources and preprocessing

The data utilized in the present study predominantly encompasses criminal offense data, extreme climate data, and social-economic data from diverse counties and cities in Hunan Province spanning the period from 2014 to 2018 (specific sources and access links are summarized in Table 1). The criminal offense data are derived from China Judgments Online. Since the implementation of the “Regulations of the Supreme People’s Court on the Publication of Judgments by People’s Courts on the Internet” in 2014, China Judgments Online has become an important source for obtaining crime data that is both publicly transparent and relatively complete [23]. This study compiled crime statistics for each county and city by carefully counting the number of publicly available criminal judgments issued by local courts across various cities in Hunan Province between 2014 and 2018. We note that while this is a public platform, consistent access to China Judgments Online from outside mainland China may occasionally be subject to network restrictions.

**Table 1:** Statistical table of provincial scale data sources.

Type	Data	Description	Data Source
Criminal offense data	Criminal offense data of counties and cities in Hunan Province from 2014 to 2018	Statistical values of criminal offenses in counties and cities in Hunan Province	China Judgments Online https://wenshu.court.gov.cn/
Meteorological data	China heavy rain dataset (2001–2019)	0.1° × 0.1°	Scientific Data Bank https://www.scidb.cn
Socio-economic data	Population, per capita GDP, total agricultural output value, etc.	Statistical data of socio-economic indicators of counties and cities in Hunan Province	Hunan Statistical Yearbook https://tjj.hunan.gov.cn/
Administrative boundaries	Hunan administrative boundary vector data	Hunan Province; county/district boundaries	Institute of Regional Sustainable Development, Hunan University of Science and Technology

The judicial data utilized in this study are all sourced from anonymized judgment documents publicly released in accordance with the law on the China Judgment Online platform. All personal information (such as names and ID numbers) was desensitized at the time of the original data release. The research process strictly complies with the “Personal Information Protection Law of the People’s Republic of China” and the relevant provisions of the Supreme People’s Court regarding the disclosure of judgment documents. Only non-sensitive fields, including case type, time, and location, were subjected to statistical analysis, without any extraction or utilization of personal information.

The climate data utilized in this context is sourced from the China Rainstorm Dataset, spanning the period from 2001 to 2019. This dataset is founded on rainfall measurements collected by the GPM satellite. This dataset provides various indicators, such as rainstorm volume and duration, at a spatial resolution of 0.1° × 0.1°. The study extracted the extreme climate data of rainstorms by subsetting the original dataset within the administrative division of Hunan Province. To tackle the challenge presented by the low resolution of raster data, the nearest neighbor interpolation method was utilized to resample the clipped data. Subsequently, zonal statistics were performed for each county and city, thereby acquiring the extreme climate indicator data, such as rainstorm duration and volume, for each respective entity. The social and economic data are sourced from the Hunan Statistical Yearbook, which is published by the Hunan Provincial Bureau of Statistics. The researchers involved in the study selected social and economic indicators, such as population density, per capita GDP, and total agricultural output value. The original data was then sorted and calculated by the researchers, who also generated social-environmental factor data to fulfill the research needs. The resultant data provides crucial social and economic background support for the study of the relationship between extreme climate events and criminal offenses.

## 3. Research methods

### 3.1 Standardized crime intensity index

The Standardized Crime Intensity Index (SCII) is a metric that has been designed to evaluate temporal variations in criminal activity. In this study, the SCII was applied to investigate crime patterns in Hunan Province. Originally proposed by Liu Lin et al. [24], the SCII refines and improves upon the location quotient, providing a more accurate measurement of crime intensity over time. This index reflects the relative level of crime intensity within a specific period by comparing the crime density of that period with the average crime density throughout the entire study period. The calculation formula is as follows:


$$SCII=\frac{C_{i}/T_{i}}{\left(\sum_{i=1}^{n}C_{i}/\sum_{i=1}^{n}T_{i}\right)}$$ (1)

where *C*_*i*_ represents the number of criminal offenses during period *i, T*_*i*_ denotes the number of basic time units contained in period *i*, and *n* is the total number of periods under consideration.

The interpretation of the index is as follows: When SCII > 1 indicates that the number of cases in that period exceeds the average level for the study period; when SCII = 1 denotes equality with the average; and when SCII < 1 signifies that the case count is below the average. This index effectively captures the changes in the intensity of criminal offenses over different time periods, providing valuable insights into the temporal dynamics of crime.

### 3.2 Local Getis-Ord Gi* index

To study the local correlation of criminal offenses, this paper applied the local Getis-Ord Gi* index [24,25]. The local Getis-Ord Gi* index serves as a measurement of local spatial autocorrelation founded upon the distance weight matrix. It is capable of detecting the existence of high-value or low-value clusters of criminal offenses in local regions. High-value clusters are denoted as hot spots, and low-value clusters as cold spots. The formula is:


$$Gi*=\frac{\sum_{j}\left(w_{ij}x_{j}\right)}{\sum_{j}x_{j}}$$ (2)

The standardized score *Z*(Gi*) is calculated as follows:


$$Z(Gi*)=\frac{\left[Gi*-E(Gi*)\right]}{\sqrt{var(Gi*)}}$$ (3)

Where, *x*_*i*_ is the attribute value of feature j; *w*_*ij*_ is the spatial weight between feature *i* and feature *j*; *n* is the total number of features. *E*(Gi*) is the mathematical expectation; var(Gi*) is the coefficient of variation. According to the value range distribution of *Z*(Gi*), different crime hot and cold spots were determined in different regional units, with ±2.58, ± 1.96 and ±1.65 as the grading criteria to divide the crime into extremely significant cold spot area, significant cold spot area, cold spot area, insignificant area, hot spot area, significant hot spot area and extremely significant hot spot area.

### 3.3 Grey correlation analysis

To explore the spatial correlation between extreme climate and crime, as well as the social environmental factors influencing crime, this paper employed the grey correlation analysis. The grey correlation analysis is a multi-factor statistical analysis method that calculates the grey correlation degree to describe the degree of correlation between the reference sequence and each related factor [26]. This paper takes the data of 122 counties and cities in Hunan Province from 2014 to 2018 as an example. It establishes the reference sequence and comparison sequence, standardizes the data using the mean method, and then calculates the correlation coefficient and correlation degree. The formulas [26] for the correlation coefficient and correlation degree are:


$$\xi_{k}=\frac{{min}_{i}\;{min}_{k}|x_{i}\left(k\right)-x_{\text{0}}\left(k\right)|+\rho \;{max}_{i}\;{max}_{k}|x_{i}\left(k\right)-x_{\text{0}}\left(k\right)|}{|x_{i}\left(k\right)-x_{\text{0}}\left(k\right)|+\rho \;{max}_{i}\;{max}_{k}|x_{i}\left(k\right)-x_{\text{0}}\left(k\right)|}$$ (4)

$$\gamma_{i}=\frac{\text{1}}{n}\sum_{i=1}^{n}\xi_{i}\left(k\right)$$ (5)

In formula (4), $X_{\text{0}}=\left\{x_{\text{0}}\left(k\right),k=1,2,......,n\right\}$ represents the reference sequence, $X_{i}=\left\{x_{i}\left(k\right),k=1,2,......,n\right\}$ represents the comparison sequence, $\xi_{k}$ represents the correlation coefficient of $x_{\text{0}}$ and $x_{i}$ at point *k*, ρ represents the resolution, 0 < ρ < 1, it usually takes the value of 0.5. In formula (5), the closer the $\gamma_{i}$ value is to 1, the stronger the correlation degree becomes. Here, *n* represents the number of data points in the comparison sequence.

### 3.4 Panel data model

To analyze the impact process and mechanism of extreme climate on criminal offenses, this paper employed the static panel data model. The static panel data model, a method that integrates the advantages of cross-sectional data and time series data [27], can address the spatiotemporal heterogeneity of research objects and reduce the collinearity between variables. Its basic form [25] is:


$$y_{it}=\alpha_{i}+\beta_{it}x_{it}+\mu_{it},i=1,2,...,N;t=1,2,...,T$$ (6)

Where, *i* represents each evaluation unit, *t* represents the time dimension, $\alpha_{i}$ is a constant term, $x_{it}$ is an explanatory variable, $\beta_{it}$ is a regression coefficient, $\mu_{it}$ is a random disturbance term. The static panel data model has three types: mixed effect model, fixed effect model and random effect model, which differ in whether they consider the fixed or random effects of individuals and time.

To ensure temporal consistency between crime and rainfall measurements, we aligned all variables at the county-year level. Specifically, each criminal case was assigned to a county and year according to its recorded date in the court document, and annual crime counts were constructed accordingly. Extreme rainfall indicators were calculated from daily precipitation records and aggregated to the same county-year units, ensuring that crime and rainfall measures correspond to identical temporal windows in the panel dataset.

## 4. Results and analysis

### 4.1 Spatiotemporal distribution characteristics of criminal offenses in Hunan Province

#### 4.1.1 Temporal distribution characteristics of crimes.

**Fig 1:**
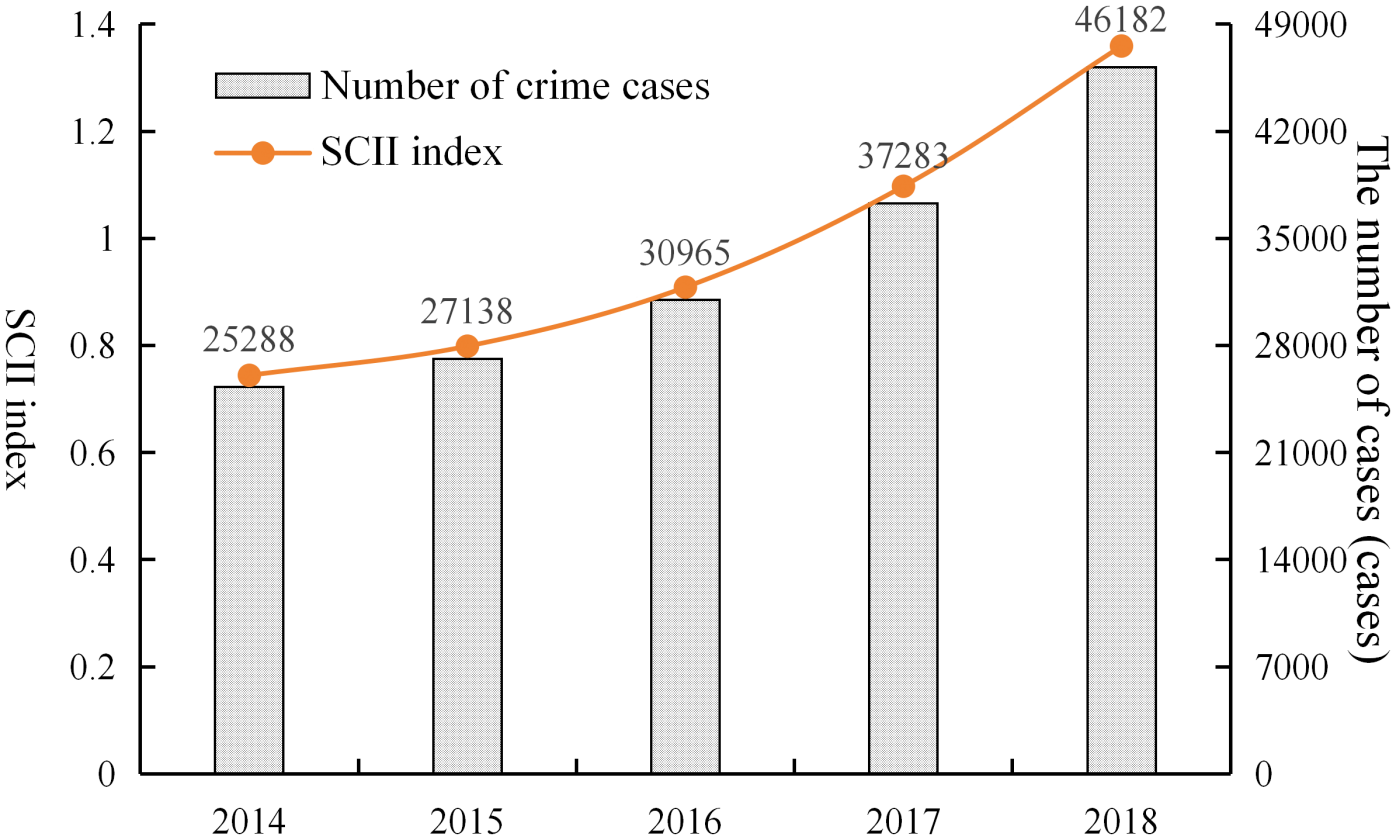
Number of crime cases and SCII index in Hunan Province from 2014 to 2018.

Fig 1 illustrates the trends in the number and intensity of criminal offenses in Hunan Province from 2014 to 2018. The standardized crime intensity index (SCII) is represented by a line graph (left vertical axis), while the number of criminal cases is displayed as a bar graph (right vertical axis). The figure demonstrates that the number of criminal cases in Hunan Province increased annually over the study period, and the growth rate showed a gradual upward trend. The SCII, defined as the ratio of criminal cases to the population, was less than one during the period from 2014 to 2016, and greater than one during the period from 2017 to 2018. This means that the level of criminal offenses in Hunan Province was lower than the five-year average from 2014 to 2016, but higher than the five-year average from 2017 to 2018. Despite the ongoing development of society, the improvement of China’s legal system, and the improvement of people’s living standards, the number of criminal cases has been increasing. This trend suggests that factors beyond general socio-economic development may also be involved, which motivates further examination of environmental influences such as extreme rainfall.

#### 4.1.2 Spatial aggregation characteristics of crimes.

**Fig 2:**
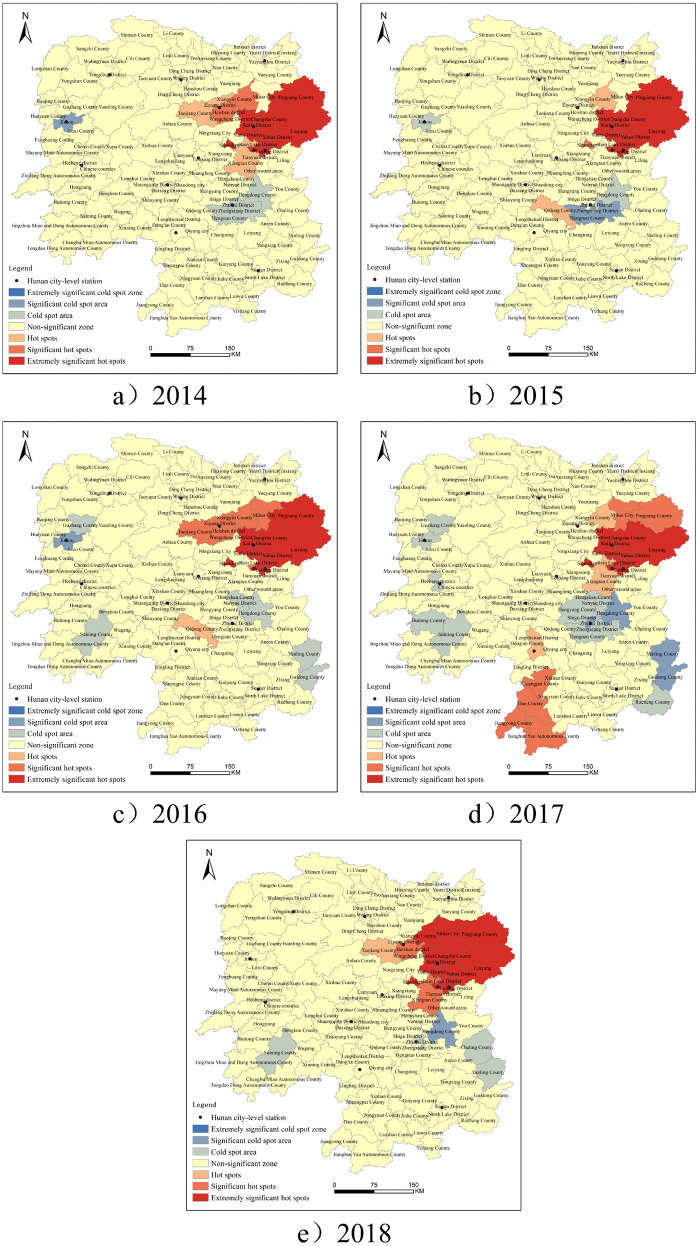
Analysis results of Gi* index of criminal cases in Hunan Province from 2014 to 2018.

Using the “Hot Spot Analysis (Getis-Ord Gi*)” tool in ArcGIS, the cold and hot spot areas of the spatial distribution of crimes in various counties and cities in Hunan Province were analyzed based on the Gi index. The Gi statistics were divided into 7 levels from high to low, namely, extremely significant cold spot areas (99% confidence level), significant cold spot areas (95% confidence level), cold spot areas (90% confidence level), non – significant areas, hot spot areas (90% confidence level), significant hot spot areas (95% confidence level), and extremely significant hot spot areas (99% confidence level), as shown in Fig 2. As can be observed from the figure, during the period from 2014 to 2018, the number of extremely significant hot spot areas for criminal offenses was the highest among all types of areas in various counties and cities of Hunan Province. In contrast, the numbers of significant hot spot areas, hot spot areas, cold spot areas, and significant cold spot areas were relatively lower, and no extremely significant cold spot areas were observed.

In terms of spatial distribution, the extremely significant hot spot areas, significant hot spot areas, and hot spot areas were mainly concentrated in the urban area of Changsha City, as well as the eastern and surrounding counties and cities. These regions are marked by relatively high levels of economic development and dense populations. They tend to have a large share of their economies driven by the service sector, experience fast urban growth, and exhibit strong social mobility. These socio-economic characteristics, specifically dense population flows and high – level commercial activity, have the potential to augment the likelihood of certain types of crime, as indicated in urban criminology research [2]. The areas with cold spots, especially the most prominent ones, were mostly located along the western and southeastern edges of Hunan Province. These areas have a relatively low level of economic development, a small population density, a high proportion of agricultural output value in the regional GDP, a relatively single industrial structure, less socio-econoic activities, and fewer crime opportunities.

In 2017, all the key crime pattern zones in Hunan Province, specifically the prominent hot spots, general hot areas, cold spots, and notable cold zones, witnessed an increase. By 2018, three additional critical hot spot areas had emerged. From 2014 to 2018, multiple regions within Hunan Province consistently exhibited high levels of criminal activity. These encompassed 14 key areas such as Liuyang City, Changsha County, Wangcheng County, and districts such as Kaifu, Yuelu, Furong, Yuhua, Hetang, Shifeng, Yuetang, Tianyuan, Lusong, and Tianxin. Throughout the research period, the pattern of the areas in Hunan Province where crimes are most prevalent exhibited certain fluctuations. Despite these changes, the areas identified as major hotspots remained relatively consistent, predominantly situated in regions characterized by robust economic activity and high population density. This inclination towards clustering underscores the crucial role played by economic, social, and demographic factors in shaping the distribution of criminal activities.

A comparison of the distribution of crime hot spots and cold spots across different years reveals that in 2017, there was an increase in the number of significant hot spots, hot spots, cold spots, and significant cold spots within Hunan Province (Table 2). Besides, in 2018, three new areas became extremely significant hot spots. Between 2014 and 2018, a number of counties and districts continuously showed extremely significant hot spots associated with criminal activity, amounting to 14 locations.

**Table 2:** Quantity statistics of local cold and hot spots (unit: number).

Year	Extremely significant cold spots	Significant cold spots	Cold spots	Hot spots	Significant hot spots	Extremely significant hot spots
2014	0	1	6	3	2	15
2015	0	2	3	2	1	15
2016	0	1	7	1	6	15
2017	0	5	12	4	6	15
2018	0	1	6	1	2	18

It is important to note that the spatial patterns revealed in Fig 2 reflect only adjudicated criminal cases. Thus, the identified hotspots may also partly indicate regions where law-enforcement capacity is stronger and criminal cases are more effectively detected, reported, and prosecuted. Conversely, areas identified as “non-significant” do not necessarily imply low crime occurrence, as underreporting or limited policing resources may reduce the likelihood of cases entering the judicial process.

### 4.2 Analysis of the impact of extreme rainfall events in Hunan Province on criminal offenses

#### 4.2.1 Spatial correlation analysis of rainfall and crime.

**Fig 3:**
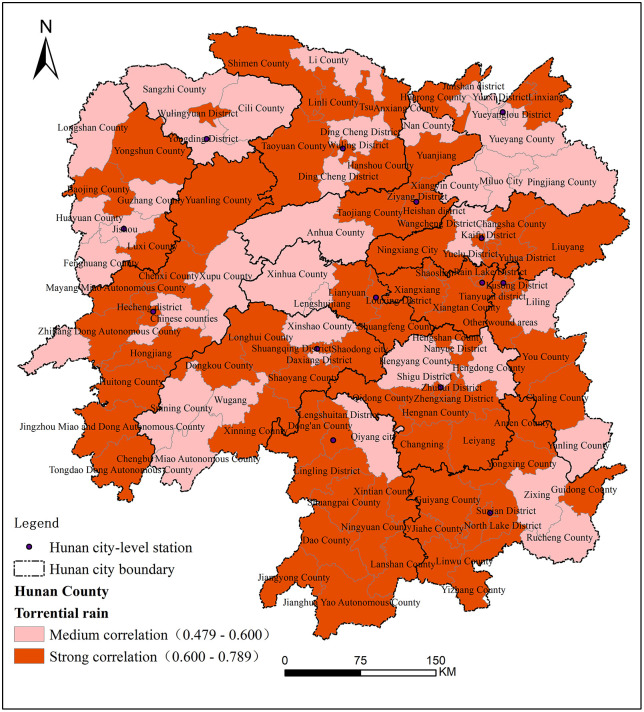
Spatial distribution of gray correlation degree between criminal offenses and rainstorm in Hunan Province from 2014 to 2018.

To investigate the spatial heterogeneity in the impact of extreme climate on criminal disturbances, this study employs the grey correlation analysis method based on the criminal offense data of various counties and cities in Hunan Province from 2014 to 2018. Two indicators, specifically rainstorm duration and rainstorm volume, were selected to represent extreme rainfall events. Subsequently, the degree of correlation between rainstorms and criminal offenses in each county and city of Hunan Province was calculated. The results of the correlation degree were spatially processed and divided into four levels: low correlation (r ≤ 0.3), medium correlation (0.3 < r ≤ 0.6), high correlation (0.6 < r ≤ 0.8), and very high correlation (0.8 < r ≤ 0.9), as shown in Fig 3.

The degree of correlation reflects the strength of rainstorms’ impact on criminal disturbances. As shown in Fig 3, the correlation coefficients between rainstorm events and crime rates in Hunan Province during 2014–2018 range from 0.479 to 0.789, indicating that extreme rainstorms had a substantial influence on criminal activity over this period. Overall, the regions in Hunan can be primarily categorized into high-correlation and medium-correlation areas. Specifically, regions such as Yueyanglou District, Hengyang County, Hengdong County, Liling County, and Qiyang County experienced relatively lower impacts from extreme rainstorm events. In contrast, areas including Ningxiang County, Dongkou County, Longhui County, Hongjiang City, and Baojing County exhibited higher sensitivity to rainstorm-induced crime disturbances. Notably, these highly affected areas tend to have a greater proportion of agricultural output in their regional GDP. Heavy rainfall has the potential to augment the economic pressure on rural households through the disruption of agricultural production. This mechanism has also been proposed in previous research regarding climate-induced income shocks and crime [3,16,17]. This is consistent with previous research indicating that weather-induced crop failures reduce household income, which may in turn compel individuals to engage in criminal activities to meet basic living needs [25]. When basic expenses cannot be met through legitimate means, the risk of criminal behavior increases. These results provide important insights into how extreme weather events can threaten public security, particularly in rural and agriculturally dependent regions.

#### 4.2.2 Analyzing the effects of extreme weather events on crime using panel data models.

This paper employs the panel data model to analyze the influence of extreme climate and various social environmental factors on crime, utilizing the county-level panel data of criminal offenses in Hunan Province spanning from 2014 to 2018.

(1) Panel Data Model Construction

This paper constructed regression models with the number of criminal offenses as the dependent variable and the extreme rainfall climate factors as the independent variables. Additionally, it incorporated population density, population mobility, per capita GDP, employment ratio, urban-rural income ratio, male-female population ratio, tertiary industry ratio and education level as control variables to control the influence of social environmental factors other than extreme climate on crime. The model formula is presented as follows:


$$CC_{it}=\alpha_{\text{0}}+\alpha_{\text{1}}EW_{it}+\alpha_{\text{2}}X_{it}+\omega_{i}+\nu_{t}+\varepsilon_{it}$$ (7)

Where, i represents the number of cross-sections, i.e., 122 counties and cities in Hunan Province, t is the year, $\alpha_{\text{0}}$ is the intercept term, $CC_{it}$ is the number of crimes, $EW_{it}$ is the extreme climate factors, $X_{it}$ represents a series of control variables, $\omega_{i}$ indicates individual effects, $\nu_{t}$ indicates time effects, $\varepsilon_{it}$ is the random error term, $\alpha_{\text{1}}$ is the regression coefficient of extreme climate on crime.

(2) Panel Model Regression Results

To investigate the impact of extreme climate on criminal offenses, this paper developed two different regression models, namely the random effects model and the fixed effects model. The random effects model assumes that individual effects are independent of explanatory variables, whereas the fixed effects model assumes that individual effects are correlated with explanatory variables. To select a suitable model, this paper employed the Hausman test [25] to compare models. The null hypothesis of the Hausman test is that the random effects model is more appropriate, and the alternative hypothesis is that the fixed effects model is more appropriate. If the P-value of the test is less than 0.05, then the null hypothesis is rejected and the fixed effects model is selected; otherwise, the null hypothesis is accepted and the random effects model is utilized. The test results are presented in Table 3.

**Table 3:** Panel model selection tests.

	Model	Test type	Statistic	P-value	Conclusion
**Heavy rain**	Model I	Hausman test	−0.093	1.000	Random effects model
	Model II	Hausman test	33.126	0.000***	Fixed effects model

Note: ***, **, and * represent the significance levels of 1%, 5%, and 10%, respectively.

As shown in Table 3, without adding control variables (Model I), the regression results for extreme rainfall climate factors and crime number used the random effects model. After incorporating control variables (Model II), the fixed-effects model was employed for the regression analysis of rainfall climate factors and the number of crimes. At the same time, this paper also employed the time fixed effects model (Model III) for comparison, to consider the time characteristics of different years. Dell and Olken et al. pointed out in their study [28] that if social and economic control variables that are directly or indirectly affected by climate are added to the model, this may lead to an underestimation of the impact of climate on crime. Therefore, to avoid over-control problem, this paper constructed models for both cases without adding control variables and adding control variables respectively, which strengthened the robustness of the conclusion. The regression results are shown in Table 4.

**Table 4:** Regression results of panel data model between rainstorm and crime.

Variables	Model I (Random effects model)		Model II (Fixed effects model)		Model Ⅲ (Time fixed effects model)	
	Regression coefficient	P	Regression coefficient	P	Regression coefficient	P
**Const**	151.845	0.001***	−445.324	0.521	−90.969	0.887
**Duration of heavy rain**	0.59	0.017**	0.356	0.010**	0.374	0.027**
**Rainfall amount**	−0.114	0.498	−0.061	0.619	0.052	0.86
**Population density**	—	—	0.135	0.118	0.02	0.252
**Population mobility**	—	—	−0.288	0.045**	0.418	0.000***
**Per capita GDP**	—	—	0.008	0.000***	0.002	0.000***
**Employment ratio**	—	—	20.779	0.701	−93.377	0.561
**Urban-rural income ratio**	—	—	−3.616	0.953	58.972	0.387
**Male-female population ratio**	—	—	−18.116	0.975	−218.186	0.696
**Tertiary industry ratio**	—	—	728.267	0.016**	457.206	0.001***
**Education level**	—	—	−178.128	0.21	29.804	0.086*
**R** ^**2**^	0.219		0.563		0.827	

Note: The dependent variable is the number of crimes; ***, **, and * represent the significance levels of 1%, 5%, and 10%, respectively.

(3) The Impact of Extreme Rainfall on Criminal Offenses

Table 4 presents the panel data model regression results regarding the relationship between extreme rainfall events and the number of criminal offenses. As shown in Table 4, Model I reveals a significant positive association between heavy rainfall duration and criminal offenses (p = 0.017), while rainfall intensity shows no significant effect (p = 0.498). This implies that the duration of heavy rain is a crucial factor affecting criminal offenses, while rainfall amount is not. After incorporating control variables (Model II and Model III), there is still a significant positive correlation between the duration of heavy rain and the number of criminal offenses (p < 0.05). In contrast, there remains no statistically significant association between rainfall amount and crime (p > 0.1). This implies that the inclusion of control variables neither alters the direction and magnitude of the impact of extreme rainfall on criminal offenses nor gives rise to the issue of over-control.

Comparing individual fixed effects model (Model II) and time fixed effects model (Model III), it is evident that the duration of heavy rain exerts a significant positive influence on crime in both models (p < 0.05). Moreover, the time fixed effects model has a better fit, with an R-squared value of 0.827, higher than the individual fixed effects model’s 0.563. This indicates that the impact of extreme rainfall on criminal offenses exhibits both temporal heterogeneity and spatial heterogeneity. In terms of temporal heterogeneity, under the premise of fixed regions, the extent of impact of extreme rainfall on crime varies in different years. For spatial heterogeneity, due to the large differences in population, economy and other social environments among 122 counties and cities in Hunan Province, the disturbance of extreme rainfall on crime also varies in different counties and cities. The regression coefficient for the duration of heavy rain in Model III is 0.374, significant at the 5% level, with a confidence interval ranging from 0.212 to 0.536.

The coefficient for population mobility among the control variables is positive and statistically major, which suggests a strong link between higher mobility and an increase in criminal cases. This suggests that regions characterized by high levels of population mobility often experience weakened social networks and reduced community cohesion, which may lower the perceived risk of detection and increase opportunities for criminal activity. Previous studies have explained the higher propensity for crime among the floating population from an economic perspective: such populations often face more challenging labor market conditions and possess more limited or insular social networks. As a result, in comparison with local residents, their opportunity cost of engaging in criminal activities is generally lower [29]. Both per capita GDP and the share of the tertiary industry show meaningful positive relationships, indicating that as economic development advances and the service sector expands, there tends to be a rise in criminal activity. One possible explanation is that economically developed regions attract larger inflows of external populations, which in turn increases population mobility. Economic prosperity often leads to a broader income gap, which can increase the motivations for crime and make certain targets more appealing. As a result, higher levels of wealth disparity tend to be associated with a rise in criminal activity [30]. Educational achievement often exhibits a distinct correlation with criminal case rates, suggesting that higher levels of education can influence legal outcomes. This may reflect the so-called “crime-expansion effect” of education, whereby individuals with higher levels of human capital have greater opportunities and access to more resources. As a result, they may possess advantages in committing certain types of high-skill crimes, such as fraud [31].

(4) Robustness Test

To ensure the reliability of the research results, this paper also conducted a robustness test by replacing the dependent variable with the crime rate per 10,000 people. The crime rate per 10,000 people refers to the number of criminal cases that occur among 10,000 people, which can reflect the crime level and crime risk of different regions. The robustness test results are presented in Table 5. In the fixed effects model that accounts for time, the coefficient of heavy rain duration and the crime rate are both positive and statistically significant at the 5% level (p < 0.05). This aligns with our expectations, indicating that longer periods of heavy rainfall tend to be associated with higher crime rates. Overall, these findings suggest that our results are consistent and remain reliable regardless of the specific dependent variable used.

**Table 5:** Robustness test results of the regression model.

Variables	Heavy rain (Time fixed effects model)		High temperature heat wave (time fixed effects model)	
	Regression coefficient	P	Regression coefficient	P
**const**	−9.822	0.389	−7.608	0.069*
**Duration of heavy rain**	0.007	0.026**	—	—
**Rainfall amount**	0.002	0.727	—	—
**Population density**	0	0.17	−0.001	0.031**
**Population mobility**	−0.002	0.135	0.003	0.018**
**Per capita GDP**	0	0.001***	0	0.000***
**Employment ratio**	0.567	0.843	5.403	0.006***
**Urban-rural income ratio**	1.765	0.148	−2.383	0.028**
**Male-female population ratio**	6.112	0.539	−4.583	0.035**
**Tertiary industry ratio**	9.44	0.000***	5.181	0.069*
**Education level**	0.224	0.466	0.165	0.791
**R** ^**2**^	0.664		0.975	

Note: The dependent variable is the crime rate; ***, **, and * represent the significance levels of 1%, 5%, and 10%, respectively.

There is a clear correlation between heavy rainfall and crime rates. When extreme weather events occur, the number of crimes tends to increase. This is consistent with the existing research findings in domestic and international literature. For instance, Stevens et al. argued that the occurrence of extreme climate would lead to an increase in property crimes, violent crimes, and other types of crimes [32].

## 5. Discussion and policy recommendations

Our analysis shows a positive association between prolonged heavy rainfall and county-level crime, whereas rainfall intensity is not statistically significant. This pattern suggests that it is the duration-driven disruption—rather than short-lived spikes in intensity—that is more consequential for crime. These findings are consistent with Routine Activity Theory [8]: sustained rainfall can disrupt daily routines and informal guardianship, reshaping crime opportunities (e.g., shifting activities indoors and altering exposure to certain offenses). They also align with prior evidence that extreme weather can affect social stability [27,32,33]. The non-significant effect of intensity may reflect that many intense events are localized and brief, and thus less likely to produce enduring changes in socio-economic conditions [16–17]. Interpretation should nevertheless consider the limited study period (2014–2018) and potential unobserved factors such as county-level policing capacity, enforcement intensity, and informal support systems.

Spatial results further indicate strong heterogeneity: rainfall-related crime sensitivity is higher in counties with greater agricultural dependence (e.g., Ningxiang, Dongkou, and Longhui). In these areas, heavy rainfall can damage crops and reduce yields and incomes, intensifying economic strain and increasing crime risk [3,4,34], similar to observations in other settings such as Taiwan [14]. While we control for key county-level socio-economic covariates (e.g., GDP per capita, unemployment, and urbanization), these results imply that rainfall is not the sole driver of crime dynamics and that context-specific factors may still matter.

Taken together, our findings refine the application of Routine Activity Theory and General Strain Theory [9] in Hunan’s climate-disruption context. The stronger role of rainfall duration highlights prolonged routine disruption and sustained livelihood strain as key pathways, while the marked spatial heterogeneity suggests these mechanisms are moderated by local socio-economic structure (e.g., agricultural dependence and mobility). This extends the use of Routine Activity Theory/General Strain Theory beyond predominantly urban settings and underscores the need to explicitly incorporate regional characteristics when applying these frameworks to climate–crime research [35,36].

These insights have practical implications. For agriculture-dominant counties (e.g., Dongkou), prioritizing post-disaster livelihood recovery and targeted relief may help buffer income shocks and associated strains; impact-based early-warning systems that account for rainfall duration could further reduce disruption (IPCC, 2022). For urban areas, given the positive association between population mobility and crime, strengthening community-based security management and social cohesion in high-mobility neighborhoods [8,13,29], alongside infrastructure resilience measures [13,16,17], may support stability during extreme rainfall disruptions.

Several limitations merit note. This study focuses on heavy rainfall and does not examine other climate stressors (e.g., heat, drought, or cold waves), and it analyzes aggregate crime rather than differentiating by offense type. In addition, some potential mediators/moderators (e.g., public safety capacity and local institutions) are not directly measured. Crime statistics are derived from court judgments, capturing only formally adjudicated cases and potentially reflecting enforcement heterogeneity across counties. Finally, the 2014–2018 period may not fully capture longer-term climate–crime dynamics.

## 6. Conclusion and outlook

This study examines the influence of extreme rainfall on crime in Hunan Province from 2014 to 2018 by combining spatiotemporal hotspot analysis with grey correlation assessment and panel econometric models. The principal findings are as follows:

(1) Crime increased during the period from 2014 to 2018, and persistent hotspots were concentrated in Changsha’s urban districts and surrounding counties (i.e., the Changsha-Zhuzhou-Xiangtan urban agglomeration), indicating clear spatial clustering.
(2) Grey correlation results indicate a moderate to strong correlation between heavy rainfall indicators and crime (correlation coefficients: ranging from 0.479 to 0.789), implying that disruptions associated with rainfall are relevant to crime dynamics, especially in counties that are dependent on agriculture.
(3) Panel-model estimates further identify rainfall duration, rather than intensity, as a robust predictor of crime: a 1% increase in rainfall duration is associated with a 0.374% increase in crime, whereas rainfall intensity does not show statistical significance. The relationship between rainfall and crime also exhibits temporal and spatial heterogeneity across years and counties.
(4) Socio-economic context moderates the relationship between rainfall and crime: higher population mobility, lower per capita GDP, and differences in industrial structure are correlated with more pronounced crime responses to heavy rainfall, which underscores the importance of place-based vulnerability.

Future research could expand upon this work by incorporating other climate extremes (e.g., heatwaves, droughts, and cold waves), differentiating effects according crime type, and incorporating key mediating/moderating factors (e.g., policing capacity and social support). Nonlinear methods (e.g., machine learning) might also contribute to capturing complex interactions and enhance predictive performance for climate-related public safety risk management.

## Supporting information

S1 Data S1 Raw Data. This is the original data1 required to replicate the manuscript’s results. S2 Raw Data. This is the original data1 required to replicate the manuscript’s results.
(ZIP)

S1 Dataset The county-level crime statistics dataset. (XLSX)
